# From medicinal plant to noxious weed: *Bryonia alba* L. (Cucurbitaceae) in northern and eastern Europe

**DOI:** 10.1186/s13002-019-0303-6

**Published:** 2019-05-09

**Authors:** Monika Kujawska, Ingvar Svanberg

**Affiliations:** 1 Institute of Ethnology and Cultural Anthropology, University of Łódź, Lindleya 3/5, 90-131 Łódź, Poland; 2 Institute for Russian and Eurasian Studies, Uppsala University, Box 514, 754 22 Uppsala, Sweden

**Keywords:** Historical ethnobotany, Booklore, Plant folklore, Folk medicine, Ornamental plants, Ethnoveterinary practice

## Abstract

**Introduction:**

White bryony, *Bryonia alba* L., is a relatively little known plant in the history of folk medicine and folk botany in eastern and northern Europe. The main aim of this article is to bring together data about *Bryonia alba* and to summarise its cultural history and folk botanical importance in eastern and northern Europe. Nowadays, this species is considered at best as an ornamental plant, and at worst as a noxious weed. However, ethnographic and historical sources show that it used to be of magical, medicinal and ritual importance in our part of Europe.

**Methods:**

A diachronic perspective was chosen in order to outline and analyse the devolution and changes in the use of *B. alba*, in the course of which we take into account the social, ecological and chemical aspects of the usage of this plant. We have therefore traced down and analysed published sources such as ethnographical descriptions, floras, linguistic records and topographical descriptions from northern and central-eastern Europe, particularly Scandinavia, Baltic States, Germany, Poland, Belarus, Ukraine and the Balkan Peninsula. The analysed material is presented and discussed within the biocultural domains that developed in the interaction between human societies and *Bryonia alba*.

**Results and discussion:**

*Bryonia alba* has many folk names in northern and central-eastern parts of Europe: some of them refer to its medicinal properties, life form, odour, or toxicity; others to its possession by the devil. As we learn, *Bryonia alba* was an inexpensive surrogate for mandrake (*Mandragora officinarum* L.) and sold as such in the discussed parts of Europe. The folklore and medicinal properties ascribed to mandrake were passed on to white bryony due to an apparent resemblance of the roots. In ethnographic descriptions, we find a mixture of booklore, i.e. written traditions, and oral traditions concerning this species. Some of this folklore must have been an alternative stories spread by swindlers who wished to sell fake mandrake roots to people.

**Conclusions:**

Plant monographs and reviews of particular species tend to concentrate on the botanicals, which might have great useful potential. White bryony presents a precisely opposite example, being a plant that used to be of medicinal relevance and was furnished with symbolical meaning, and has nowadays preserved only its ornamental value among some urban and rural dwellers of northern Europe. Nonetheless, it might be considered as a part of the biocultural heritage in old, well-preserved gardens. It is still used as a medicine in some parts of the Balkan Peninsula.

## Introduction

The European white bryony, *Bryonia alba* L., is a relatively little known plant in the history of folk medicine and folk botany in eastern and northern Europe. However, in 1792, the botanist Carl Fredrik Hoffberg wrote enthusiastically about *Bryonia alba*, which was then either naturalised or planted in Sweden. A deep hole cut into the roots after they had been cut off evenly at ground level was filled with juice after a day or so. This juice cured oedema and was employed in the treatment of intestinal worms, convulsions and headaches. Slices of the fresh root were applied on bruises, and an ointment made from the root was used in cases of pneumonia. *Bryonia alba* was also utilised as a climbing plant on round pole fences and walls, and was good for covering wooden walls, portals and gazebos [1].

This perennial vigorous vine has been mentioned as a medicinal plant in many herbals in northern Europe since medieval times, was part of pharmacopoeias, could be used for ornamental purposes and was well known in the local folklore. It still had multipurpose uses in the eighteenth century, but its popularity decreased during the nineteenth century, and for the last hundred years, the plant has been mostly forgotten or regarded as a weed in northern and eastern Europe [2].

**Fig. 1:**
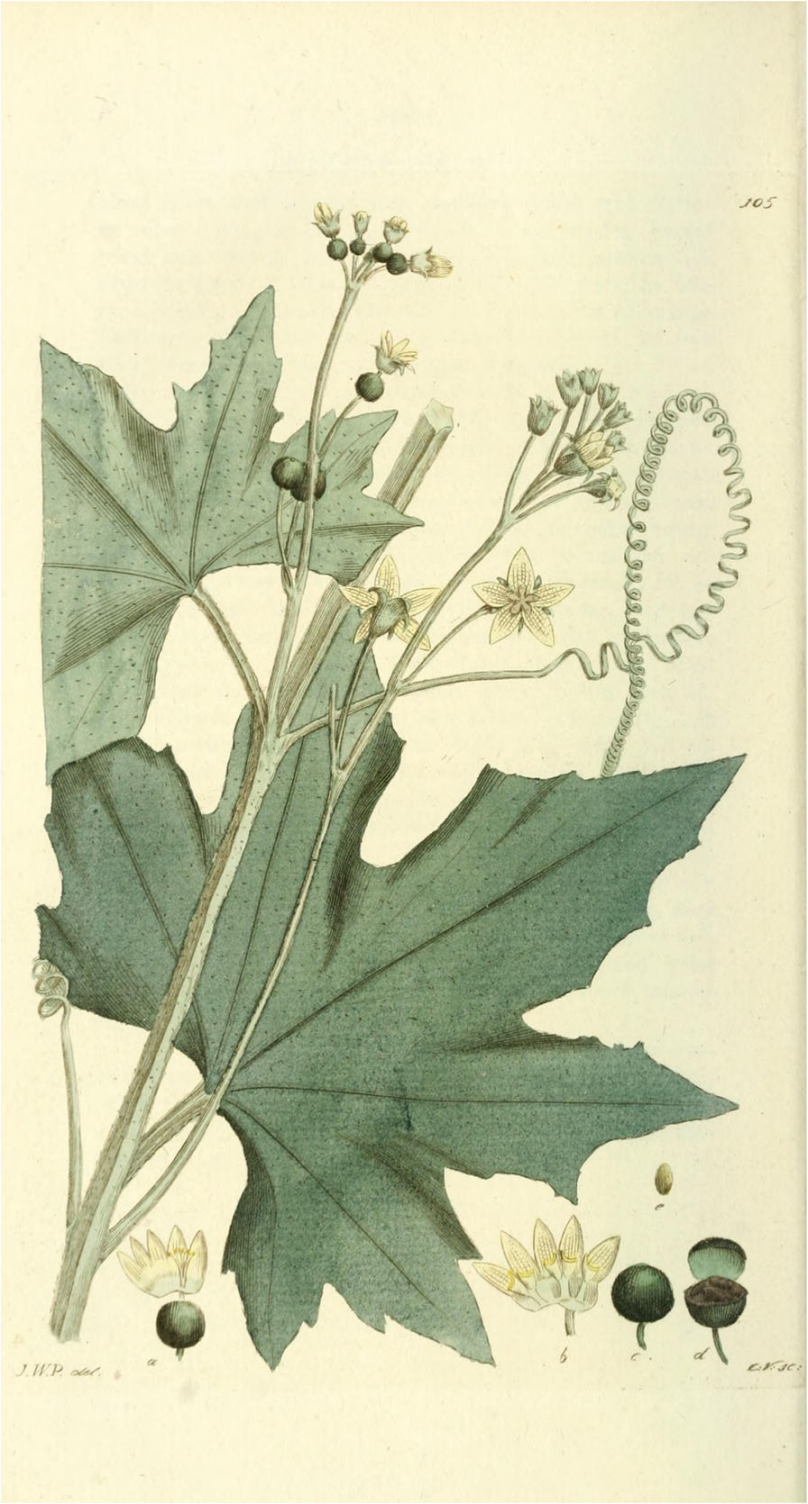
*Bryonia alba* L. (Cucurbitaceae) illustration from Johan Wilhelm Palmstruch, Svensk botanik, (Stockholm1803)

The main aim of this article is to bring together data about *Bryonia alba* and to summarise its cultural history and folk botanical importance in central, eastern and northern Europe, especially within the regions referred to by ethnologists as the Scandinavian, West Slavic and Balkan cultural areas, although some data from beyond these areas will be included as well (Fig. 1) [3]. Nowadays, *Bryonia alba* is considered at best as an ornamental plant, and at worst as a noxious weed. However, ethnographic and historical sources show that it used to be of magical, medicinal and ritual importance in our parts of Europe [4]. Our interest in this plant, therefore, is in analysing its progressive devolution and depreciation in these parts of Europe, in the course of which we will take into account the social, ecological and chemical aspects of its usage.

## Material and methods

This is a mainly historically oriented piece of research, and we have therefore traced down and analysed published sources such as ethnographical descriptions, floras, linguistic records, topographical descriptions and travel narratives [5, 6]. An intense literature search for references to the use of *Bryonia alba* was performed from northern and central-eastern Europe, particularly Scandinavia, the Baltic States, Germany, Poland, Belarus Ukraine, Romania and parts of the Balkan Peninsula. National borders have shifted in recent centuries, and extensive demographic changes have occurred within the research area. A diachronic perspective was chosen in order to outline and analyse the devolution and changes in use of this plant. The examined material is presented and discussed within biocultural domains that have developed in the interaction between human societies and *Bryonia alba*. It has not always been easy to distinguish between wild and cultivated plants. In most cases, the results will only give us a fragmentary insight into the biocultural domains that developed between human beings in our part of Europe and the taxon *Bryonia alba*. It is necessary to emphasise that the knowledge and local utilisation of this species may have included other domains, which have not left any traces in the kind of sources we have used.

**Fig. 2:**
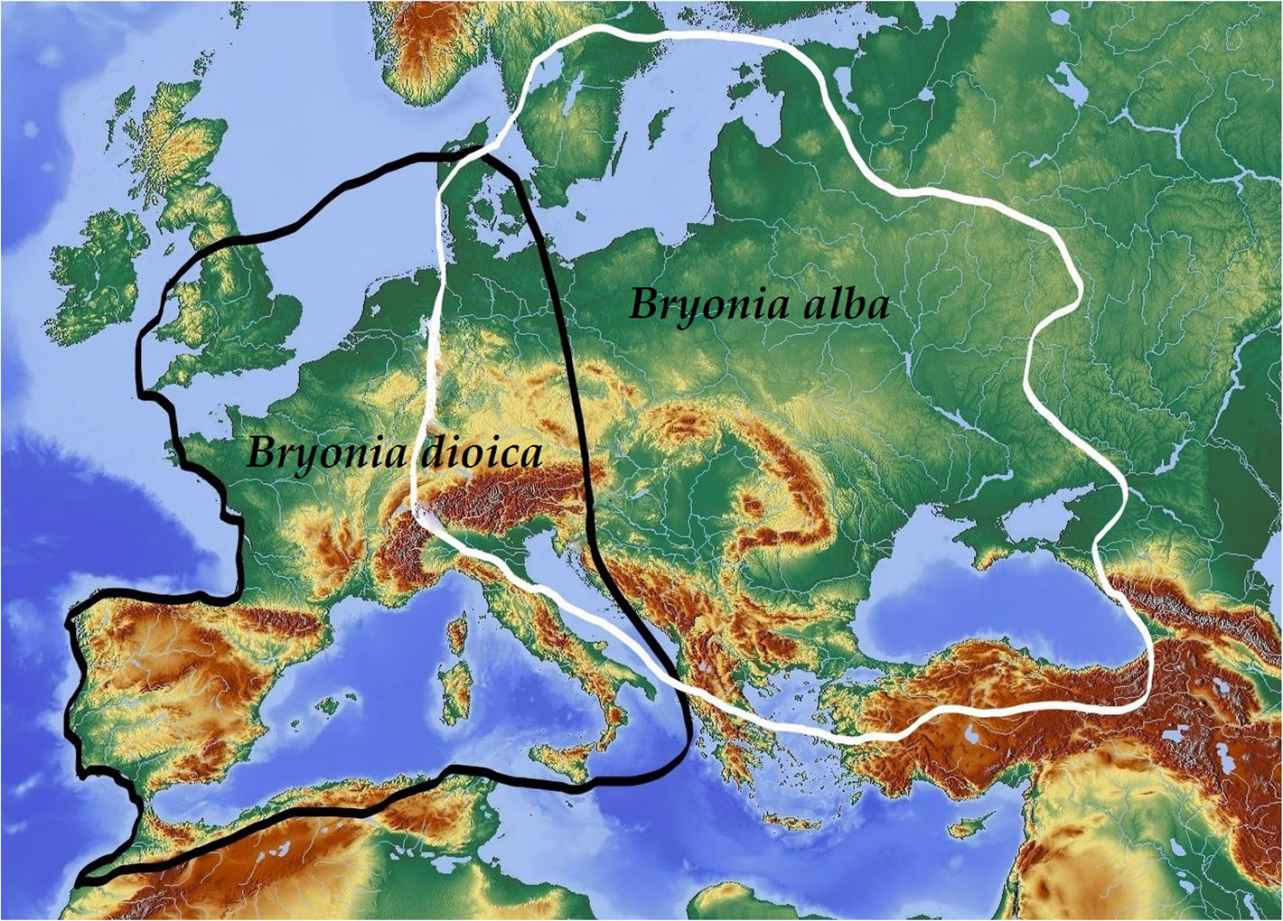
Geographical distribution of *Bryonia dioica* and *B. alba*. Adapted from Global Biodiversity Information Facility (gbif.www); Artdatabanken (artfakta.artdatabanken.se); and Volz and Renner in *Taxon* 58(2); 2009. Map was drawn by Hele Kiimann, Uppsala University

In central, eastern and north Europe, both *B. alba* and *B. dioica* Jacq. (Syn. *Bryonia cretica subsp. dioica* (Jacq.) Tutin) coexist (Fig. 2). *B. alba* most probably extended to north Europe from the southeast, while *B. dioica* reached north Europe via Spain and France [7]. Nonetheless, *B. dioica* is rarer than *B. alba* in these regions. The ethnographic and historical sources of these parts of Europe ascribe nearly all cultural uses to *B. alba* (apart from the Balkans, where also *B. dioica* has been endowed with diverse cultural applications). For example, in Polish ethnographic sources, only Szulczewski [8] and Milewska [9] mentioned medicinal uses of *B. dioica*. However, there are many similarities between these two species when it comes to folk beliefs, medicinal use and rituals in Great Britain and Western Europe [10–13]. During the review of written sources for this contribution, we did our best to separate the mentions on *B. dioica*, then describe and analyse those that referred to *B. alba* [14]. The authors intend to come back to *B. dioica* in another paper, which is covering German-, Latin- and Celtic-speaking, Europe as well as the Slavic-German borderlands [4, 11, 12].

## Biology, ecology and distribution

*Bryonia* genus (Cucurbitaceae) contains 12 species, which prosper in the floristic regions of the Mediterranean, Irano-Turanian and in part the Holarctic floral kingdoms. All *Bryonia* species have their origin in the Irano-Turanian region in Asia [7]. Some species have a narrow distribution and are endemic to certain areas, others have a wide range, are poorly morphologically distinct from one another and are often themselves polytypic: *B. dioica*, *B. cretica* L., *B*. *multiflora* Boiss. and Heldr., *B*. *monoica* Aitch. and Hemsl., *B. aspera* Steven ex Ledeb. and *B. alba* [15].

*Bryonia alba* has, as British botanist Charles Jeffrey points out, the largest and most northerly distribution from all *Bryonia* species, ranging from about 8°E in Western Europe to the southern Urals and northern Iran in the east [15]. The ranges of *Bryonia alba*, especially the northern limits, may likewise have been expanded by human activity. Scandinavian sources mention the introduction of the species already during medieval times, most probably due to monastic activities. It was previously cultivated in home gardens, hedges and stonewalls, but later escaped into the natural environment and become a naturalised plant, still very anthropogenic adapted [7, 16–18]. Geobotanical and archaeobotanical studies from central and eastern Europe identify this species as archaeophyte [19] or as kenophyte (alien plants that arrived in this area after the conquest of America) [20].

**Fig. 3:**
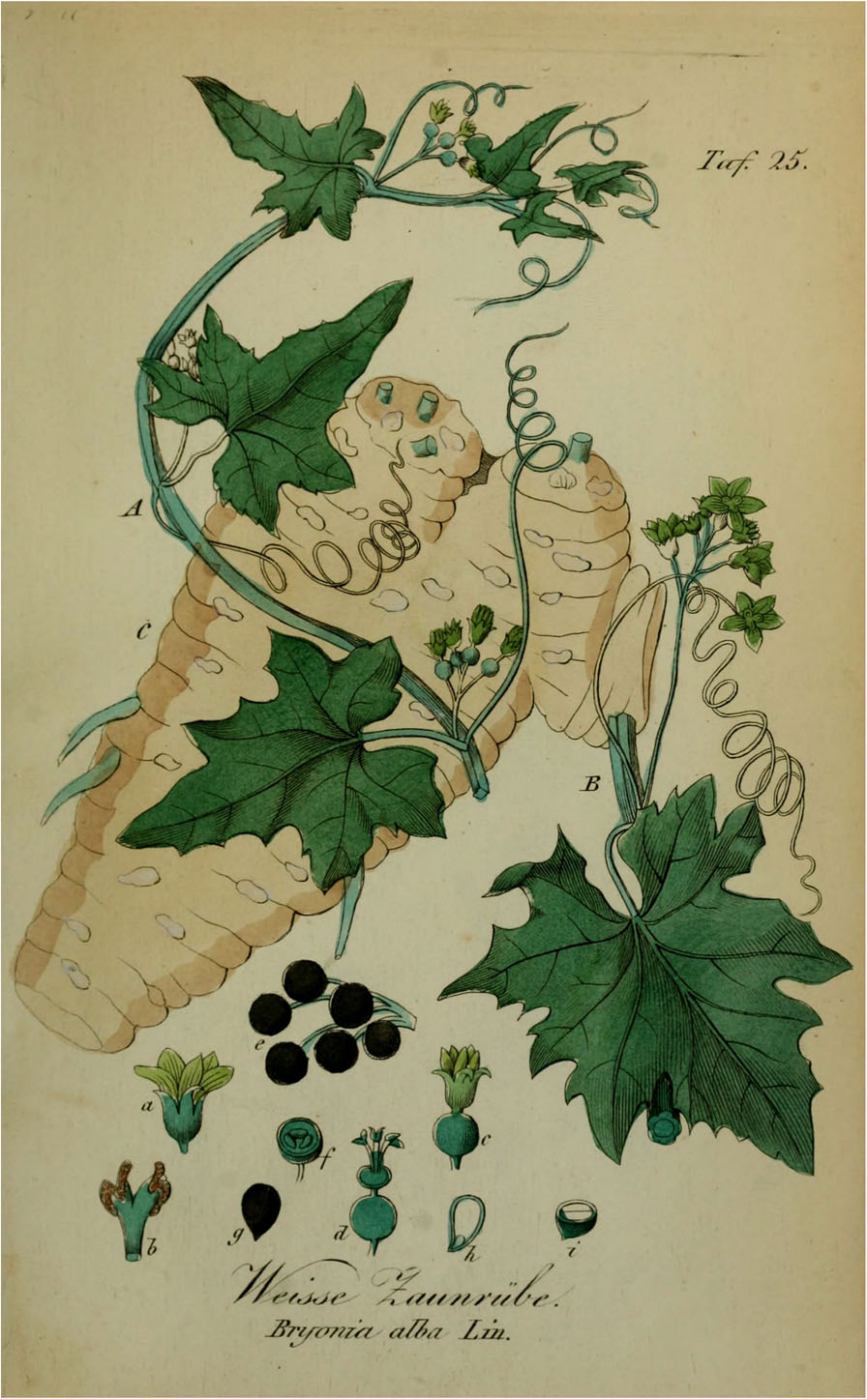
Root and berries of *Bryonia alba*. Illustration from Eduard Winkler, *Sämmtliche Giftgewäckse Deutschlands* (Leipzig 1854)

White bryony is a herbaceous perennial vine [21]. Like other *Bryonia* spp., it occurs on well-drained soils, such as sand dunes or dry riverbanks. The leaves are triangular or heart-shaped and broadly toothed. The blossoms are greenish-white in colour. The stigma is glabrous. The fruit is never red, ripening directly from green to black, which is one of the characteristic taxonomic features of *B. alba*. It has a water-storing tuber-like root, yellowish-white in colour (Fig. 3). The species is monoecious, i.e., both male and female flowers are found on the same plant. The plant is unusual in being a diploid apomict (clonal reproduction), with the capacity for sexual reproduction [15]. Although monoecious in northern Europe, this species in the southeastern part of its range, i.e. Macedonia and Turkey, often appears to be dioecious. This has led to its confusion with *B. dioica* in these areas [15].

*B. alba* has been valued as a medicinal plant since ancient times, and there is no doubt that it has been widely disseminated and introduced with regard to this usage [2, 4]. In northern and central-eastern Europe, the root was often sold and used as a substitute for mandrake (*Mandragora officinarum* L.) [22]. This is already mentioned by Renaissance botanist Hieronymus Bock in his herbal from 1539 [23]. For example, in Germany, itinerant peddlers, hawking plants, used to cut and carve roots of *B. alba* (growing in Bavaria), *B. dioica* (growing commonly in the south-west) and *Acorus calamus* L. or *Dactylorhiza maculata* (L.) Soó into human shapes. Then, they would put germinating barley grains inside the root, so the root was covered by a rough surface. Such prepared roots would be sold to people [22]. In northern Europe, swindlers were known to carve white bryony roots into a certain shape, to bury them in dry sand for some days, and then to sell them as mandrake [4, 24]. In Poland, swindlers used to sell rhizomes of *Phragmites australis* Trin. ex Steud., *Iris* sp. and *Nymphaea* sp. in addition to roots of *Bryonia alba* as mandrake [22]. Mandrake first started to lose its magical importance and then, since the eighteenth century, its medicinal relevance, hence swindles of this type occurred with decreasing intensity [22].

## Local names

*Bryonia alba* has many folk names in northern and eastern parts of Europe. The Benedictine abbess Hildegard of Bingen recorded the phytonym *Stickwurtz* ‘stitch root’ already in 1160, and it is known from many herbals and handbooks since. The name refers to its medicinal uses against stitches in the side. Other local and folk names not only refer to medicinal application, but also to magical properties of the plant, as well as its shape, status and growing habit.

The plant is known as *hundrova* in Sweden, which means ‘dog’s turnip’—with the first word understood pejoratively [25]. This is probably due to its unpleasant odour. The name was already mentioned in 1638 in a plant list by the botanist Johannes Franckenius in Uppsala [26]. Danish and German folk names have the same meaning, *hunderoe* 'dog’s turnip' [18] and German *Hundsrüben* [27]. The folk names in Finnish *koirannauris* 'dog’s turnip' and Estonian *koeranairis* 'dog’s turnip', respectively, are probably calques from Swedish or German [28, 29]. The Lithuanian name also refers to dogs. There it is called *šunmolūnas,* ‘dog’s friend’ and *šùnobuolas,* ‘dog’s pumpkin’ [27, 30]. In Germany and Denmark, it has been known as *Teufelrübe* and *djævelroe* ‘devil’s turnip’, and in south Russia as паралисьзная репа ´paralysing turnip’, most probably referring to its toxicity [2, 18, 31]. Croatian names also refer to turnip, e.g. *repa divja* ‘wild turnip’ [32], as well as one of Romanians’ phytonyms: *napul dracului* ‘devil’s turnip’ [33].

That the plant was regarded as an alien, i.e. non-native, species already in medieval times, is shown by its ancient Swedish name *valsk rova* ‘foreign turnip’ (ca 1520) [34] and its Danish equal *walske roffue* by Christian Petersen in 1533 and Smid in 1546 [35, 36]. Linnaeus recorded *tysk rova,* ‘German turnip’, in 1749 in Skåne, the southernmost province of Sweden [37].

Nowadays, the plant is commonly known as *Zaunrübe* (known since 1534) in German, which refers to its location choice (climbing on fences). Similar names exist in German and Swedish as well, *Heckenrüb,* ‘hedge turnip’, and *gärdsgårdsrova* ‘fence turnip’, were recorded for the first time in 1803. [17, 18]. Phytonyms referring to its habitat are also given in Czech, for instance *živý plot,* ‘living fence’ and *kořen zaplotní kový* (‘on fence growing plant’) [27], and can be found in Romanian, *mutătoáre en poame negre* ‘climbing vine with blackberries’ [38].

The great importance of this plant in Romanian folk medicine is reflected in one of the folk names, *împărăteasă* ‘empress’ [38]. A large number of folk appellatives refer to illnesses, for example Russian сонное зелье ‘sleep potion’ and змиева ягода ‘snake berry’ [31], German *Gichtrübe,* ‘gout turnip’, and *Gichtwurtz,* ‘gout root’. The known Swedish equivalent of this latter German name is *giktrot,* ‘gout root’. It is also known as *jichtna řipa* (‘gout root’) in Sorbian [39]. All the mentioned names indicate the root to be a characteristic or useful part of the plant [4]. In Denmark and Norway, the denominations are *galdebær* and *gallbær* respectively. This refers to the bile green colour and bitter taste of the berries [40]. Other Danish names are *vild drue,* ‘wild grape’ and *vild græskar,* ‘wild pumpkin’ [18, 40]. Some Ukrainian phytonyms also contain derogative to the fruits’ meaning, such as *гадючi ягоди* ‘viper berry’ and *вовчi ягоди* ‘wolf berries’ [41].

In Poland, the common name of this species, *przestęp biały,* is most likely derived from its folk version: “its name is after stepping down of the divine commandment” [42]. The name *przestęp* was used throughout Poland, reflecting a strong consensus on the plant’s name [43]. Because of its similarity to the mandrake root, it was sometimes confused with this plant: “the root of bryony is to be completely similar to a child, and therefore has the head, eyes, ears, belly” [44]. Due to some similarity with the fruit of deadly nightshade [*Atropa belladona* L.], it was confused with this plant too [45]. In the east of Poland (i.e. after shift of the borders, nowadays western Ukraine), it was called similarly, *perestup* or *neczipaj zilje* [46]. The name *perestupen*’ is still used in Russian and Ukrainian [47], in Belarus it is known as пярэступ белы [38], and *poćel* in Upper Sorbian [27]. Varhol [48] has recorded similar names, *prestupnica* and *perestupen*’, among the Carpathian Ruthenian minority in Slovakia (Prešov region). The Czech name—*posed*—also comes from *posednutý*, which means possessed. The Slovakian equivalent—*posed biely*—is very similar and refers to the same origin—the possession of the root by the devil [48, 49]. Other Slovakian name *zemská tekvica* ‘earth pumpkin’ has its equivalents in Croatian—*buča divja* or *tikva divja* ‘wild pumpkin’, Serbian—*debelo tikva* ‘fat pumpkin’ and in Bulgarian—*diva tikva*. In all these languages, the prototype plant is the pumpkin [49–52]. It is also known as ‘wild pumpkin’ in Romanian—*cucurbătă sălbatică* [33]. Interesting names were recorded in Croatia, which evoke medicinal uses and properties, which have no resemblance in other aforementioned phytonyms—*kuge trava*—‘plague herb’ and *bluščec črnojagodasti*. *Bljušt, blušč* and *bluščec* origin from an old Slavonic name meaning ‘to vomit’ and *črnojagodasti*—‘having black berries’ [32]. Hence, the latter Croatian name actually reassures us that the discussed species is *B. alba*.

## Results and discussion

### Folk beliefs and ritual uses

The role of white bryony in folk beliefs and Catholic Church rituals has been well described in Polish ethnographic sources from the nineteenth and the beginning of the twentieth century. For Epiphany (6th of January), a root of *B. alba* and *Juniperus communis* L. were blessed in church and used later in cows’ fumigation, when a cow suffered from an udder infection (central-western Poland) [53]. It used to be blessed in bouquets on Assumption Day [15th of August] in eastern Poland among the Polish-speaking population (nowadays western Ukraine), but this practice was already abandoned by the nineteenth century, due to a belief that the demon lived in the root [54] (Table 1). In the Kraków area, this practice survived until the beginning of the twentieth century, but only witches blessed white bryony on Assumption Day [43]. The blessed root was used by witches to steal milk from other people’s cows. Łuczaj recorded that the habit of blessing white bryony during the Assumption Day in southeast Poland was still remembered by elder people in recent years [55]. White bryony was also blessed in wreaths during the Corpus Christi Octave, along with other herbs, such as *rozchodnik* (*Sedum telephium* L.), *piwonia* (*Paeonia* sp.), żegawka (*Urtica urens* L.), *macierzanka* (*Thymus pulegioides* L. or *T. serpyllum* L.) and *bez* (*Sambucus nigra* L.) among others. The whole wreath was used in cows’ fumigations before and after calving [53, 56]. It had also an apotropaic function and was used during stormy weather, especially to ward off thunder. Nonetheless, it is difficult to indicate the role of *B. alba* in these practices [56]. This plant was also used during sepulchral rituals—it was placed in a coffin, as a “pillow” for a dead person (east Poland) [57].

**Table 1:** *Bryonia alba* L.—categories of uses based on historical and ethnographic material

Category of use	Use [reference]	Region and country
Folklore^a^	Blessed in bouquets on Assumption Day [43, 54, 55]	South-eastern Poland
Folklore^a^	Blessed in wreaths during Corpus Christi [53, 56]	Poland
Folklore^a^	Sepulchral rituals—placed in a coffin, as a “pillow” for a dead person [57]	Eastern Poland
Folklore^a^	Apotropaic for people and domestic animals [56]	Poland
Folklore^a^	Used by witches to harm people and their cattle [54, 58, 59]	Poland
Folklore^a^	Bringing luck plant, endowed with transformative powers [54, 58]	Poland
Folklore^a^	Brings luck to the household [31]	Southern Russia
Folklore^a^	Digging the plant required putting some offering (bread, coins), in return a spirit who lived inside would not get irritated and seeking revenge [48, 60, 61]	Poland, Ukraine
Folklore^a^	Folklore of love and courtship [58]	Poland
Folklore^a^	Folklore of love and courtship [24]	Lower Rhine, Germany
Folklore^a^	Substitute for mandrake (*Mandragora officinarum*) [22]	Germany, Poland
Folklore^a^	Substitute for mandrake (*Mandragora officinarum*) [18]	Denmark
Food (emergency)	Starch-rich roots were recommended for baking emergency bread [62–64]	Finland, Sweden
Food (poison)	Berries can be highly toxic [65]	Croatia
Medicinal (folk)	Wounds, ulcers [9, 66–68]; fruits against sore throat and oedema [66–68]	Lithuanian-Belarus borderland
Medicinal (folk)	Wounds, mixed with fat against scabies, chest pains (the root, mixed with honey and brandy), fever, rheumatism [38, 69]	Romania
Medicinal (folk)	Folk illness: *oberwanie* (an effect of lifting something heavy); *plica polonica* [70]	Poland
Medicinal (folk)	Epilepsy [71]	Denmark
Medicinal (folk)	Leaves used in contusion, bruises, bone fracture [72]	Lithuania
Medicinal (folk)	Internal parasites, abortifacient [73]	Ukraine
Medicinal (folk)	Constipation [62]	Sweden
Medicinal (folk)	Viper bites [18]	Denmark
Medicinal (folk)	Snake bites [69]	Romania
Medicinal (folk)	Deters snakes [31]	Southern Russia
Medicinal (folk current use)	purgative, diuretic, mucolytic, against dropsy, gout, lung catarrh, diarrhoea, epilepsy, wounds, ulcers [51]	Deliblato Sands, Serbia
Medicinal (folk current use)	Anti-rheumatic [74, 75]	Kosovo
Medicinal (historical)	Oedema, intestinal worms, convulsion, headache, bruises, pneumonia [1]	Sweden
Medicinal (historical)	Stitches on the side [4]	Germany
Medicinal (historical)	Stitches on the side [35, 36]	Sweden
Medicinal (historical)	Epilepsy [35, 36]	Sweden
Medicinal (historical)	Constipation; used to remove a dead foetus [36]	Sweden
Medicinal (historical)	Internal parasitic worms, laxative, aches and sores [76]	Sweden
Medicinal (historical)	Hysterical disorders, inflammation of the hands [77, 78]	Sweden
Medicinal (historical)	Pneumonia, gout [79]	Germany
Medicinal (historical)	Laxative and purgative medicine [80]	Poland
Medicinal (historical)	Dizziness, as heart tonic [81]	Poland
Ornamental	Good for covering wooden walls, portals and gazebos [1]	Sweden
Ornamental	Ornamental plant in crofters and peasant gardens during the nineteenth century [17, 76, 82–84]	Sweden
Veterinary (folk)	Used to enhance cow milking [53, 85]	South-eastern Poland
Veterinary (folk)	Blessed on St. George’s Day and used in rituals to increase fatness of the cow’s milk [86]	Upper Pčinja, Leskovačka Morava in Serbia
Veterinary (folk)	Blessed for Epiphany together with common juniper and used in cow fumigation, when a cow suffered from udder infection [53]	Central-western Poland
Veterinary (folk)	Given to pigs, cattle and sheep to prevent and cure several illnesses [38]	Romania
Veterinary (folk)	Given to domestic hogs for parasitic worms and anthrax; pigs’ prophylaxis [18]	Denmark
Veterinary (folk)	Goats’ treatment (unspecific) [87]	Sweden
Veterinary (folk)	Peasants often grew it close to henhouses as it could keep away birds of prey [4]	Scandinavia

^a^Folklore means here folk beliefs and ritual use

In parts of Serbia, *debelica* (another name for *B. alba*, ‘the fat one’) was used on Saint George’s Day (23rd of April) in household prosperity rituals. In Upper Pčinja, it was used as a waistband to gird women and buckets—“so that milk would be fat”. In Leskovačka Morava, other Serbian region, children had been girded with white bryony. Then wreaths, called *kolo*, were plaited out of it to be used for sheep milking. The day before Saint George’s Day and on that day, as well sheep were milked through these wreaths [86].

Folk tales present white bryony as an ambivalent plant – endowed with the power of both bringing good luck and happiness, and taking it from people. Due to this power, white bryony could protect people, their households and cattle, against witches’ spells and bring them wealth. On the other hand, it could harm people and their cattle. Moreover, white bryony had been endowed with transformative powers [54, 58]. The root could turn into a small child. After the transformation, it could make a (wo)man rich but it usually did so to their eventual loss:

“*Przestęp* does not want to be cultivated, it likes to escape. It comes out of the earth, when an owner notices this, he must pour it with sugared water. The owner removes bryony root usually in October during a new moon, always on Friday. After this, the root should be washed in a cold water from a river or alternatively in white sour wine (...). Then it must be put under the owner’s shirt and kept like this for three nights. White bryony must be called by its name - as if it were baptized. Then the owner must wear his root in a new white canvas shirt, made from a new piece of canvas that has not yet been worn, and he needs to bathe it every month on Friday, otherwise it would start to cry, like a small child, or even more terribly. *Przestęp* cured in such a way may grow to the size of a 3-year-old child, it starts to walk everywhere, usually to the most dangerous places, and it reveals hidden treasures in the earth. The owner will become rich inevitably, but he usually ends his life tragically and all his property is lost too, as if the curse gravitated on him and his belongings” [58].

In south Russian folklore, a dried root used to bring luck to household [31]. Slavonic folk tales also indicate that digging the roots required a special offering to the plant or the place where it grew. The person was supposed to put a few coins or a piece of bread in the same place. If one did not obey this rule though, he might get ill, or a child sitting in the bryony root might cry and harm the person [54]. Similar information comes from eastern part of Poland [60, 61]. Ukrainians associated this plant with the ability to disappear from the place where it grew and move to other places. This happened when someone dug the root out without asking the owner of the *perestupen’* for permission. The offended *perestupnica* became rotten and found another convenient place near a fence or a farm building [48].

White bryony was used in the folklore of love and courtship in Poland:

“A woman digs it out of the earth during a waxing moon at night. In the following morning, before the sunrise, she hides three pieces of the root, puts them in a new clay pot, adds some *dobra myśl* [*Origanum vulgare* L.], pours some water from a river or a mountain stream, covers it tightly so that steam would not go out and prays 3 times *Pater Noster*. When the water boils, she turns the pot on a stove, with a bare hand, eastward direction, three times only and then she puts the pot on the stove edge for a while. After 5 minutes or so, she brings the pot to boiling once again and says a prayer while turning the pot in a manner described above. Then again, she repeats this action for the last time. The water must boil three times, and the pot must be turned just three times every time the water boils. The woman must think about her lover and he will come to her” [58].

The described practice is very similar to one involving European wild ginger (*Asarum europaeum* L.) in the love and erotic lore known from Polish ethnographical literature [88]. Germans used *Bryonia alba* to make a kind of love potion from the plant. Moreover, young women in the Lower Rhine kept thin slices of the root in their shoes in order to attract men [24].

Through these examples, we learn that white bryony was a cheap surrogate for mandrake. It was used in a very similar way to the mandrake root, which is inaccessible in this part of Europe [89]. Characteristic features are the anthropomorphization of the root and the treatments performed on it, such as pouring sugar or wine over it, or wrapping it in white canvas—local materials were used instead of silk or red wine as recorded in German sources [22]. Records from Denmark also indicate this trend [18].

Was, therefore, the use of white bryony an example of booklore, i.e. the literary tradition rather than an oral one [90]? Early Polish herbals [91–93] contain a critique towards beliefs in the magical powers and human shape of the mandrake root, concentrating rather on the medicinal properties of the mandrake, which were actually very few. Therefore, there must have been an alternative folklore spread by swindlers who wished to sell fake mandrake roots to people at local markets and during church festivals. Eventually, the properties and folklore related to mandrake passed on to white bryony. Some cliché uses of *B. alba* transferred from other plants, such as *Asarum europaeum*, are also found in folk botanical descriptions [88]. In Pokuttya and Bukovina (a border region between Ukraine and Romania), among Ruthenians and Vlachs, it was deadly nightshade (*Atropa belladonna* L.) which was used as a substitute for mandrake [94]. In the Bukovina region, it was called *matraguna*. The Hutsuls from the Ivanofrankivsk area used to call *A. belladona* similarly: *matrygan* (матриган). The above-described ritualised form of collecting roots of white bryony was observed among the Hutsul and Boykos, but in respect to deadly nightshade. The Hutsuls paid earth with a coin for extracting the root, while the Boykos did this with a piece of bread and salt [94]. The botanist Karl Hölzl, who in 1861 published an exemplary, extensive report on the ethnobotany of the Ruthenians in Eastern Galicia (Galizien) and Bukovina, describes how the herdsmen and villagers feared the *Bryonia alba* and avoided it [95].

### Medicinal properties and uses

*Bryonia* has been used in both scholarly and folk medicine. It has also been used in folk veterinary medicine. Records of medicinal uses of *Bryonia* have been known for over two millennia. However, we cannot be sure whether they concerned *B. alba* or other species within the genus [14]. Most probably the first references come from Hippocrates (460–380 BC), and other mentions come from Pedanius Dioscorides’ *De Material Medica* book IV, Chap. 182 (written approx. 65 BC), or Pliny’s *Historia Naturalis* (77 BC) [7]. The ancient Greek physicians recommended the root against gout, epilepsy, paralysis, vertigo, hysteria, sores, and coughs. Dioscorides recommended it for treating burns. Hippocrates prescribed it against tetanus. Identifying the *Bryonia* mentioned in these sources is problematic; however, as is shown by Wagner, the medieval pharmacological literature sometimes mixed up *Bryonia* ssp. and *Dioscorea communis* (L.) Caddick & Wilkin [96].

*B. alba* was then mentioned in early modern herbals, such as Hieronymus Bock 1530, Leonhart Fuchs 1542, Jacobus Theodorus Tabernæmontanus 1588 and Nicholas Culpeper 1653, Jakub Haur 1693, etc. [2, 34, 81, 96]. According to Scandinavian Renaissance scholars Christiern Pedersen [35], canon of Lund Cathedral, and Henrik Smid [36], who practiced medicine in Malmö, wearing the root around one’s neck counteracted epilepsy, while boiling it in oil yielded a remedy for stitches in the side. Smid also recommended juice of the berries for constipation. The same author also mentioned that a piece of root placed in the vagina could remove a dead foetus. German herbalists in the fifteenth and sixteenth century confirmed this and thus used the name *Stickwurz* for it. Juices from the plant and berry were used to treat oedema too [27]. In a Polish herbal from the seventeenth century, *przestęp* was described as an excellent remedy against dizziness, as heart tonic and a protector against witchcraft [81]. Eighteenth-century physicians averred that if slices of the fresh root are placed against aches and sores, the effect is to cure the ailment. The root was deemed effective against parasitic worms, and it was used as a laxative [76].

Due to these numerous and diverse uses, European pharmacies long-carried the root of white bryony [97]. It was enlisted as a medicinal plant in the official pharmacopoeia in Germany, Denmark, Sweden and Poland. In Sweden, it was included as *bryoniae radix*, *bryoniae bacca* and *bryoniae semina* in the pharmacopoeias until 1817 [98]. In Denmark, *Bryoniae radix* was mentioned in the pharmacopoeia of 1772 (18]. In Poland, it was mentioned in *Pharmacopoea Regni Poloniae* from 1817 [80]. During the seventeenth century, it was considered effective against hysterical disorders. It was also used for inflammation of the hands and was therefore called *kvesrot* in the medicinal literature [77] (recorded since 1684 [78]). It was also considered effective against pneumonia, and had a long-standing reputation as active against gout [79]. White bryony had also a reputation as an effective laxative and purgative medicine [80].

According to Voltz and Renner [7], the main reason why *Bryonia* species had been so important for medicine since ancient Egypt, Greece and Rome, then rewritten in the Medieval and Renaissance periods and later on present in official pharmacopoeias, is that *Bryonia* species are rich in different cucurbitacins. These chemical compounds are biologically active and are known for strong cytotoxic and antitumor action [99], but in high doses, they can be poisonous. Separate assays have been done on *B. alba*, sometimes in combination with *B. dioica* [100–102]. Moreover, *B. alba* roots have high phenolic and flavonoid content [103]. The recognised pharmacologically medicinal activities of *B. alba* are anti-rheumatic, expectorant and anti-inflammatory [103]. Although, historical and some folk medicinal sources have mentioned *B. alba* as a remedy for epilepsy, there are not enough studies to confirm its application in epilepsy [104].

Nowadays, the tincture is used in homoeopathy, given in very small doses for the relief of joint and muscle pain. It is regarded as one of the best diuretics and has been used for relieving coughs and colds of a feverish, bronchial nature [101, 102]. There is also a renewed interest, despite concerns, in the plant within alternative medicine [105].

It is above all the root, which has been used in folk medicine, in both fresh and dried form. The usual practice was to harvest it in autumn [51]. This empirically developed local practice is supported by pharmacological findings, according to which fresh roots collected in summer time contain very toxic cucurbitacin I, whereas dried roots collected in autumn or springtime contain only glucosidases and the less toxic tetrahydrocucurbitacin I [101]. Berries can be highly toxic too. According to Pintar [65], the lethal dose for children is 15 berries, and for an adult person 40 berries consumed.

Some folk medicinal uses stay in line with the pharmacologically described properties of the species. In the Vilnius area (nowadays the Lithuanian-Belarus borderland), the leaves were applied to wounds and ulcers [9, 66]; and the fruits against sore throats and to reduce oedema where prepared in a decoction. A similar use was reported for Polish-speaking people in nowadays Belarus [67, 68]. In Romania, white bryony was also a medicine against wounds, and mixed with fat was used against scabies [38, 69]. In the central part of Poland, rural people used the root for toothache. It was helpful for dizziness and as a tonic—a plaster made of the grated root was used for this event [106]. An identical remedy and a form or registration was recorded by Haur in his herbal from 1693 [81]. White bryony had an application in several folk illnesses (or culture bound-syndromes) too. Boiled with milk, it was drunk against *oberwanie* (an effect of lifting something heavy), and against *plica polonica* [70].

In Denmark, this plant was a remedy for epilepsy, but the aerial parts were used—the juice was put in the nose [71]. In Lithuania, leaves were used externally in compresses to treat contusions, bruises and bone fractures [72].

White bryony was an important remedy in Romania against fever, chest pain (the root, mixed with honey and brandy, was taken in the morning) and against rheumatism [38].

Ukrainians gave the plant to their children in order to expel intestinal worms. According to information from nineteenth-century Ukraine, it has also been used as an abortifacient, due to its contractive effect upon the uterus [73].

Swedish peasantry in the eighteenth century consumed it in beer, for constipation, which was most probably influenced by the aforementioned herbals [62]. The Danish peasantry used the juice of the berries for the same purpose. The plant also cured viper bites [18]. Danish and Romanian ethnographic sources inform that this plant also cured viper bites [18, 69]. In southern Russia, it was used to deter snakes [31]. Therefore, we find ethnographic accounts of similar practices in three linguistically different regions of Europe.

Why has white bryony practically disappeared from folk pharmacopoeias? Nowadays, ethnobotanical studies in central-eastern and northern Europe do not mention any use for *Bryonia alba*. The only current example of uses comes from relatively isolated regions in the Balkan Peninsula. Popović and colleagues [51] recorded its use among Serbs in Deliblato Sands/Deliblatska peščara, where the root of *debelo tikva* apparently retained versatile applications as purgative, diuretic, mucolytic, against dropsy, gout, lung catarrh, diarrhoea, epilepsy, and externally for ulcers and wounds. It is still used in Kosovo, mostly by ethnic Albanians. Two local names have been recorded *stërkungulli* and *kungëlli i egër*, used as anti-rheumatic agent, squeezed and topically applied to the painful area [74, 75]. We may surmise that the decreasing interest in this species as a medicinal plant stems from its toxicity [65]. Other plants, more efficient or more available, must have replaced white bryony in the treatment of headaches, intestinal worms or constipation. Moreover, white bryony has mostly been applied externally, and as we can observe in European folk medicine, external applications are increasingly rare [107]. Moreover, since the nineteenth century, the plant has been closely associated with witches’ activity; therefore, country people preferred to turn to other medicinal plants, in order not to be labelled as users of witchcraft [45].

### Ethnoveterinary application

Polish peasants applied white bryony for the fumigation of cows, when cows could not produce milk due to the activity of witches. Witches would bathe the root of white bryony in milk in order to draw the milk from neighbours’ cattle. Ostling [59] mentions that witches also drew milk from mice, cats, rabbits and other vermin. The decoction of this herb was added to cows’ fodder to enhance milking [53, 85]. In Romania, an admixture of root was given to pigs, cattle and sheep to prevent and cure several illnesses [38]. In Denmark, it was given to domestic hogs for parasitic worms and anthrax. It could also be added to drinking water to protect pigs from diseases [18]. In Sweden, it has been used in folk medicine, especially for goats [87]. In Scandinavia, peasants often grew it close to henhouses, in the belief that it kept away birds of prey [4].

The plant was avoided as food by domestic animals due its unpleasant smell, with the exception of the goat, which could eat small amounts of it [62, 82]. However, birds can safely feed on the berries. In Sweden, birds like blackcaps, *Sylvia atricapilla* L., have been observed eating the berries during wintertime [108].

### Apparently a food plant

The dried root is rich in starch. In the eighteenth century, the starch-rich roots were recommended for baking emergency bread in Finland and Sweden [62, 63]. Still, in World War I in Sweden, it was recommended that starch be obtained from the root. It could also be used for baking emergency bread [64].

### From an ornamental plant to a common weed

Ethnographic sources contain very little information about the management of white bryony, apart from the aforementioned magical practices related to offerings. However, Butură mentions for Romania, that in order to have the plant at hand, some moved it from the fences, where it was exposed to dirt, and grew it in clean places [38]. In Scandinavia, the plant is an introduced species, often planted near villages and human settlements. It was still a popular ornamental plant in the crofters and peasant gardens during the nineteenth century [17, 76, 82–84], used to cover walls, fences and verandas. It was also considered an apotropaic agent for thunderstorms and bad weather. In the early twentieth century, it lost its popularity, but in many old gardens, it remains as a relic [1, 82, 109]. It is still found feral in urban areas [5, 16, 17, 110]. However, contemporary garden historians in northern Europe have discovered that several old garden plant relics still exist as naturalised plants, even as weeds, in urban areas [111, 112]. They are therefore considered a biocultural heritage, worthy of being cared for and preserved for the future. *Bryonia alba* is one of these old garden relics which Scandinavian garden historians try to save [113].

Nevertheless, white bryony nowadays is usually considered a weed. In many parts of the world, it has become naturalised. In North America, for instance, it started to spread as an invasive plant during the last quarter-century, and it is now classified as a noxious plant. In some US states, it is known as the *kudzu of the northwest*. The name “kudzu” most probably refers to Japanese kudzu (*Pueraria montana* (Lour.) Merr.), which was introduced to the USA in the 1880s. [114]. White bryony has also been introduced to New Zealand [7]. Therefore, we observe the anthropogenic range expansion of the species, with a simultaneous decrease in its use.

## Conclusions

Plant monographs and reviews of particular species tend to concentrate on those botanicals, which may have great useful potential or have been very important for a local population but are overlooked in wider food, medicinal or other contexts. White bryony presents a precisely opposite example, being a plant, which used to be of medicinal relevance and was furnished with symbolic meaning, yet nowadays has preserved only its ornamental value among some urban and rural dwellers of northern Europe. Nonetheless, it might be considered a part of the biocultural heritage in old, well-preserved gardens, as northern European historians claim.

As we have learnt, *Bryonia alba* was an inexpensive surrogate for mandrake and sold as such in the discussed parts of Europe. The folklore and medicinal properties ascribed to mandrake were passed on to the white bryony due to an apparent resemblance of the roots. The promotion of the rational use of medicinal plants by biomedicine and the increasingly symptomatic character of folk medicine have undermined the position of plants with “magic, human like” roots. In Scandinavia, Germany and among western Slavs, its use is obsolete today. Folk medicinal application has been preserved in the Balkan Peninsula, though in relatively isolated regions. White bryony has also lost its relevance in ethnoveterinary practice, and as emergency food. The first of these practices is rarely performed by modern farmers, and as an emergency food, this plant shares the fate of many wild edible plants of a similar kind, that are of little value in times free of bad harvest and social unrest.

It is, however, possible that the long medicinal and magical history of use of white bryony will inspire avant-garde artist and designers to find a way for artistic articulation for this plant, something that has already happened with *Monstera* spp. and other plants in recent years.
